# Imaging of renal pseudotumors in children: a comprehensive review

**DOI:** 10.1007/s00247-025-06320-4

**Published:** 2025-07-10

**Authors:** Burak Dalkıran, H. Nursun Ozcan, Berna Oguz, Mithat Haliloglu

**Affiliations:** Department of Radiology, Hacettepe University School of Medicine, Ankara, 06100 Türkiye

**Keywords:** Magnetic resonance imaging, Pediatrics, Pseudotumor, Renal lesion, Ultrasound

## Abstract

Certain renal developmental anomalies and non-tumorous lesions in children may mimic malignant tumors, leading to diagnostic challenges. These lesions, known as pseudotumors, require careful differentiation to avoid unnecessary invasive procedures. In this context, imaging techniques play a critical role in differentiating pseudotumors from malignant neoplasms. Ultrasound (US) is the preferred initial imaging modality in pediatric patients. When US findings are inconclusive, magnetic resonance imaging is commonly used as the next step. Computed tomography is less commonly preferred due to ionizing radiation. It is useful to evaluate the lesion along with the clinical and laboratory findings, and the follow-up imaging may be necessary in some cases. This review presents common and uncommon developmental, infectious, granulomatous, vascular, and miscellaneous renal pseudotumors in the pediatric population.

**Graphical Abstract:**

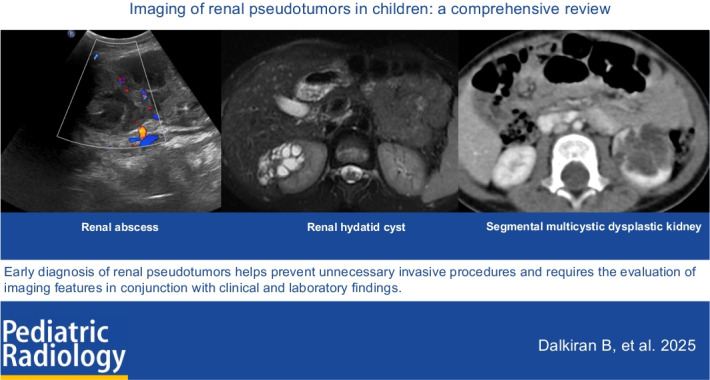

## Introduction

Pediatric renal tumors are very heterogeneous, encompassing a spectrum from tumors with low malignant potential to highly aggressive tumors. Various non-neoplastic lesions may exhibit imaging characteristics that resemble those of malignant tumors. These lesions are referred to as pseudotumors. Imaging modalities play a critical role in detecting and distinguishing these mimickers from true neoplasms.

Ultrasound (US) is often the initial imaging modality for evaluating renal lesions. In inconclusive cases, as an intermediate step prior to magnetic resonance imaging (MRI), contrast-enhanced ultrasound (CEUS) may be used to characterize renal masses. As a real-time imaging technique, CEUS is relatively cost-effective and does not expose patients to potentially nephrotoxic agents or ionizing radiation [1]. In cases where US or CEUS findings are inconclusive, MRI is usually recommended as the next step due to its lack of radiation. The disadvantages of MRI include its lengthy duration, high cost, delayed appointments, requirement for breath-holding, and the need for sedation especially under 5 years of age. The “feed and wrap” technique can be used to eliminate the need for sedation in MRI examinations of infants. In this method, feeding induces natural sleep, while wrapping helps to minimize movement artifacts. Computed tomography (CT) is less preferred due to ionizing radiation and is generally used when MRI is contraindicated or unavailable. Keeping the imaging features of pseudotumoral lesions in mind and correlating them with the clinical and laboratory findings of the patient can help prevent unnecessary biopsy and resection.

This review will discuss renal pseudotumors in children, classifying them into developmental, infectious, granulomatous, vascular, and miscellaneous renal lesions (Table 1).

**Table 1:** Classification of renal pseudotumors

Developmental	Infectious-granulomatous
Prominent column of Bertin	Focal bacterial nephritis and renal abscess
Persistent fetal lobulation	Renal tuberculosis
Dromedary hump	Xanthogranulomatous pyelonephritis
Splenorenal fusion	Sarcoidosis
Crossed fused renal ectopia	Hydatid cyst
Segmental multicystic dysplastic kidney	
Vascular	Miscellaneous
Extramedullary hematopoiesis	Scarred kidney and regeneration nodule
Arteriovenous malformation	Hemorrhagic cyst
Spontaneous subcapsular hematoma	

### Developmental renal pseudotumors

#### Prominent column of Bertin

The normal morphology of the kidneys can be disrupted due to the failure of polar parenchymal absorption, resulting in a prominent column of Bertin, which is considered a variant of normal anatomy. This condition is predominantly observed in the middle third of the kidney [2, 3].

**Fig. 1:**
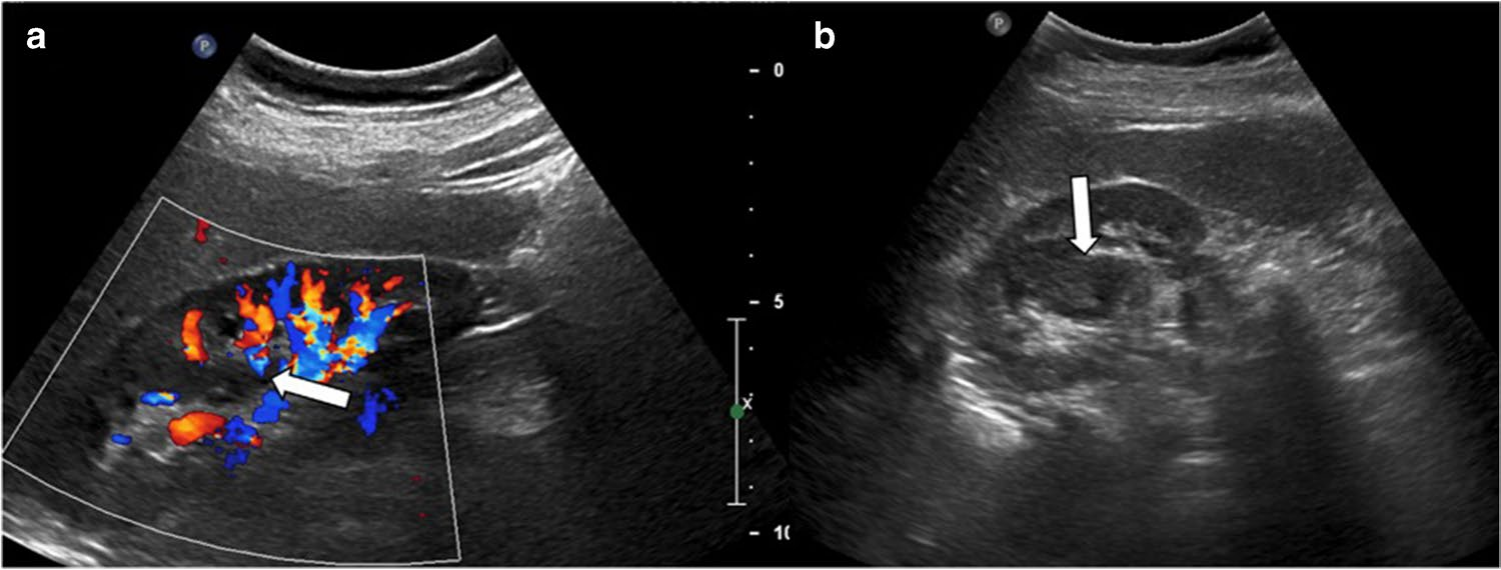
Prominent column of Bertin. A 13-year-old boy presented to the Emergency Department with abdominal pain. **a** Longitudinal color Doppler ultrasound (US) image shows that there is no abnormal internal vascularity (*arrow*) in contrast to the renal tumors. **b** Axial grayscale US image demonstrates a hypertrophied column of Bertin (*arrow*) in the transition between the upper and the middle thirds of the right kidney. Note that this area is homogeneous and has a similar echotexture to the surrounding parenchyma, and the renal contour is preserved

On US, it may appear as a mass-like extension into the renal sinus. These areas exhibit isoechoic features relative to the surrounding renal parenchyma, with hyperechoic foci representing interlobar vascular structures. Doppler US examination demonstrates a flow pattern containing arterial and venous vascular structures similar to those found in normal renal tissue (Fig. 1). Although US is often sufficient for diagnosis, in atypical cases presenting with a medullary pseudotumor appearance, contrast-enhanced US or MRI should be considered in children to exclude a true mass [2, 4, 5]. On contrast-enhanced US, CT, and MRI, the column of Bertin exhibits contrast enhancement similar to the normal renal cortex, unlike neoplasms.

#### Persistent fetal lobulation

The renal lobules are separated during the first two trimesters of fetal life by sulci that indent the renal surface. In the third trimester, these sulci disappear and the renal surface becomes smooth [6]. Incomplete fusion of these lobules leads to persistence of fetal lobulation. Focal forms may be mistaken for scar tissue or a tumor [7]. Cortical depressions between two pyramids—and not within a pyramid—help differentiate this condition from cortical scarring [8].

On US examination, the echogenicity is similar to normal renal tissue, and Doppler US shows a symmetrical, regular vascular pattern. In focal cases, further evaluation with contrast-enhanced US or MRI may be warranted. The contrast enhancement pattern in persistent fetal lobulation remains consistent with normal renal parenchyma across all phases of imaging, whereas true masses typically demonstrate different enhancement in at least one phase [9].

#### Dromedary hump

The contour of the kidney can be altered by adjacent organs, with the spleen most commonly affecting the shape of the left kidney. In this case, a focal mass appearance may develop on the lateral edge of the left kidney, which is known as dromedary hump. Due to its typical location along with echogenicity and vascularity similar to renal parenchyma, the diagnosis can often be established using US, and further imaging is generally not required [10, 11].

#### Splenorenal fusion

Splenorenal fusion occurs as a developmental anomaly secondary to the fusion of nephrogenic mesoderm and splenic primordium in the second month of gestation [7]. In the majority of cases, patients present with an asymptomatic abdominal mass; however, clinical manifestations of hypersplenism can occasionally be observed [12].

Splenorenal fusion can mimic Wilms’ tumor and renal cell carcinoma. Due to the lack of specific imaging findings, differentiation is mostly not feasible using US, MRI, or CT. Single-photon emission CT (SPECT) with 99mTc-heat-damaged red blood cells or 99mTc-colloid (sulfur or albumin colloid) is a specific method for detecting splenic tissue. In suspicious cases, fine-needle aspiration may also be performed to confirm the presence of a heterotopic spleen [13].

#### Crossed fused renal ectopia

Crossed fused renal ectopia is the second most common renal fusion anomaly after horseshoe kidney. Ectopic kidney is located on the opposite side of the ureteral insertion to the urinary bladder, and its ureter crosses the midline, unlike horseshoe and pancake kidneys. Left-to-right ectopia is more common than right to left, with a ratio of 3:1 [14].

**Fig. 2:**
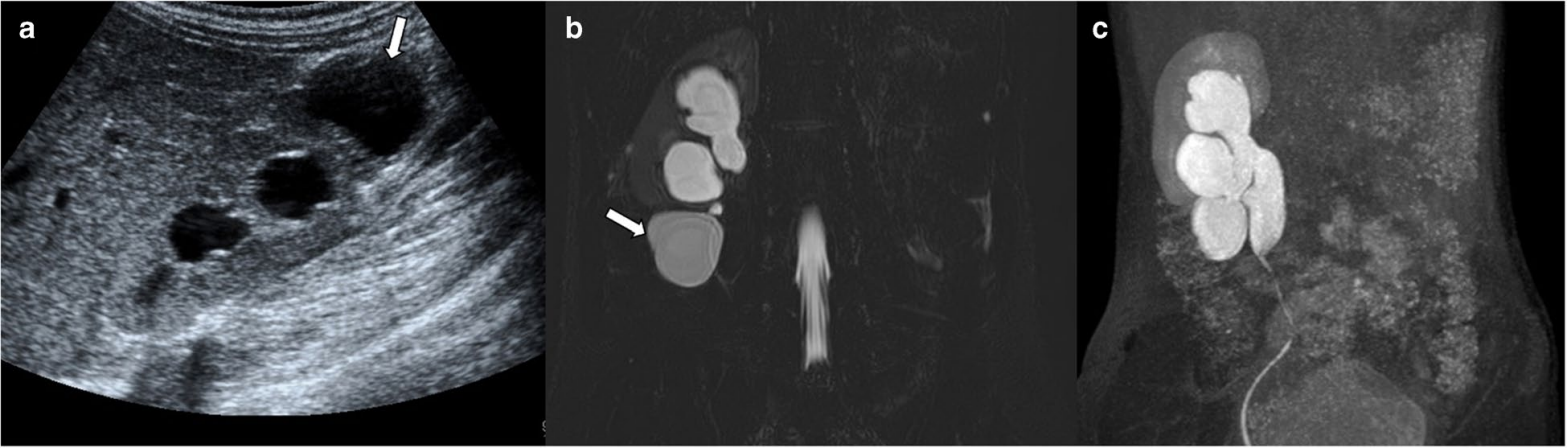
Crossed fused renal ectopia. An 8-year-old boy presented to the hospital with abdominal pain and ultrasound (US) was performed. **a** Longitudinal grayscale US image shows a thick-walled cystic lesion (*arrow*) in the inferior pole of the right kidney. The left kidney was not visualized in the left renal fossa. **b** Coronal T2-weighted maximum intensity projection (MIP) image demonstrates a cystic lesion (*arrow*) in the inferior pole of the right kidney that is not connected to the collecting system. **c** In the delayed phase, coronal post-contrast T1-weighted MIP image reveals contrast retention in the cystic lesion, indicating the dilated pelvicalyceal system of the ectopic left kidney

The diagnosis is often made using US in cases of non-involuted cross fused kidney. However, involution of ectopic kidney may mimic inferior polar solid mass on US. Sometimes, severe dilatation of the collecting system with parenchymal involution may appear as a cystic polar lesion. MRI is helpful for suspicious cases, especially in the presence of parenchymal involution. Discrimination of renal medulla and cortex is important for diagnosis, and signal intensity of non-involuted ectopic kidney is similar to normal kidney. MR urography can demonstrate the collecting system of an involuted ectopic kidney and aid in distinguishing it from a true cystic or solid mass (Fig. 2).

#### Pancake kidney

**Fig. 3:**
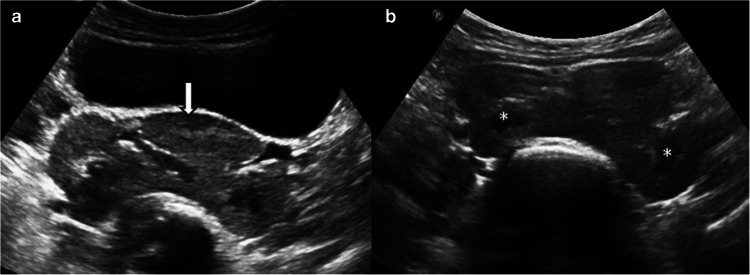
Pancake kidney. A 1-year-old girl presented to the hospital with recurrent urinary tract infections. Both kidneys were not visualized in the renal fossa. **a** Axial grayscale ultrasound (US) image shows a large, lobulated renal mass (*arrow*) posterior to the urinary bladder.** b** The medulla (*asterisk*) and cortex, exhibiting different echogenicity on US, are distinguishable, consistent with pancake kidney

Pancake kidney is a rare congenital anomaly characterized by complete fusion of the superior, middle, and inferior poles of both kidneys in the pelvic cavity. Each kidney has a separate pelvicalyceal system, renal pelvis is anteriorly placed with two short ureters that do not cross the midline and enter the normal bladder position [15]. The main feature is a large and lobulated renal mass composed of two fused lateral lobes without a septum, which may be mistaken for a pelvic mass. Diagnosis is usually made with US based on the typical renal morphology and the absence of kidneys in their expected locations (Fig. 3).

#### Segmental multicystic dysplastic kidney

Multicystic dysplastic kidney is a congenital malformation characterized by multiple non-communicating cysts of varying sizes, separated by dysplastic parenchyma. This anomaly results from abnormal and incomplete kidney development, leading to minimal or absent renal function. The segmental form is a rare subtype, comprising about 4% of pediatric cases. Notably, most cases of the segmental form are associated with duplex collecting systems [16].

Multicystic dysplastic kidney has been categorized into three distinct forms based on imaging and pathological features. The classic type is the most common form, characterized by multiple cysts of different sizes with minimal dysplastic stroma. The hydronephrotic type presents with a dilated renal pelvis surrounded by cysts. Solid cystic dysplasia consists of numerous small cysts embedded within a nonfunctional stromal component [17].

**Fig. 4:**
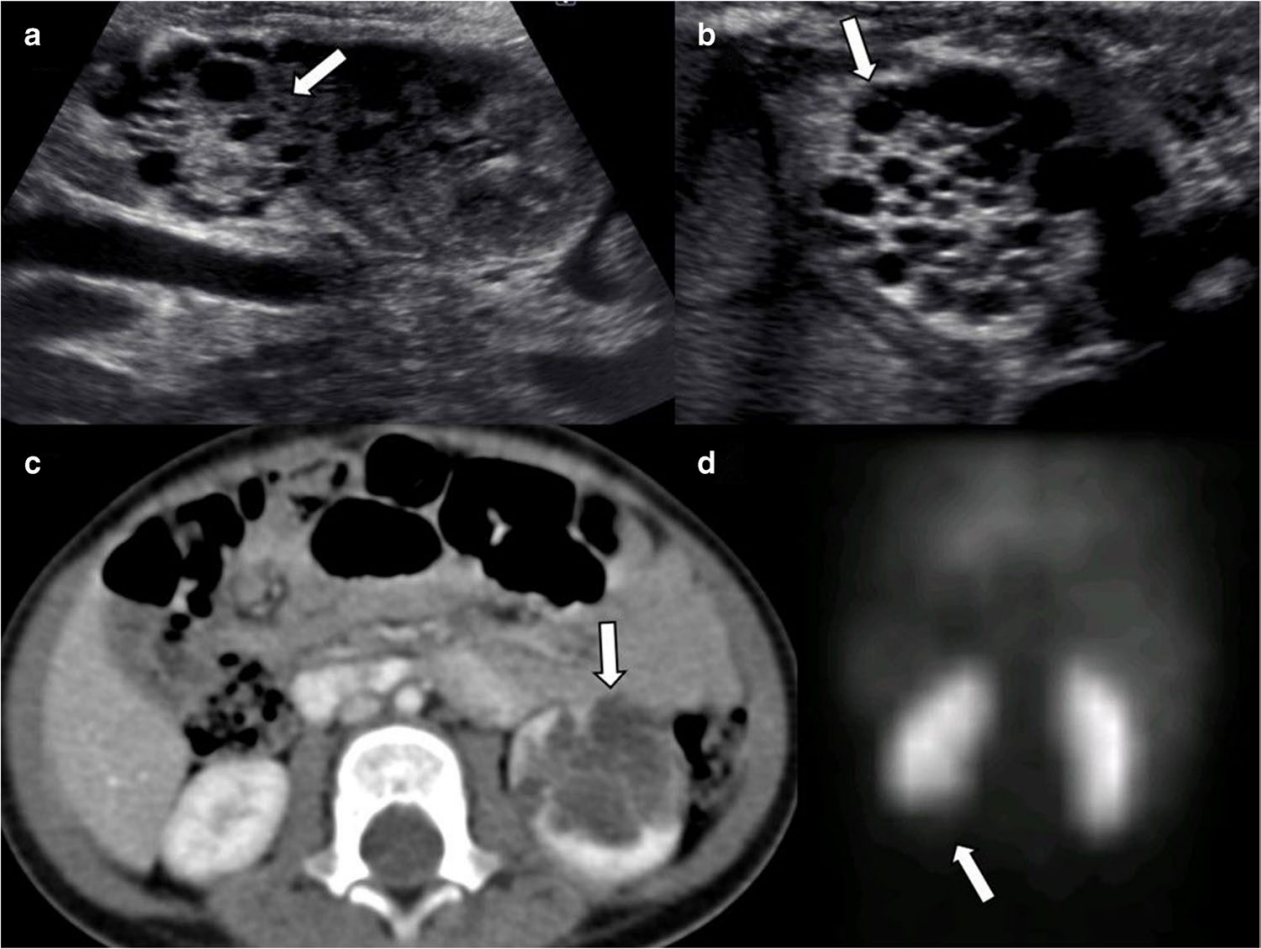
Segmental multicystic dysplastic kidney. Two girls aged 3 months (**a**,** b**) and 15 months (**c**,** d**) presented to the hospital with recurrent urinary tract infections. **a**,** b** Longitudinal (**a**) and axial (**b**) grayscale ultrasound images show a lesion (*arrows*) composed of non-communicating small cysts and echogenic stroma in the upper pole of the left kidney. **c** Axial contrast-enhanced computed tomography image reveals a multilocular cystic lesion composed of cysts of various sizes (*arrow*) in the lower pole of the left kidney. **d** Dimercaptosuccinic acid scintigraphy shows an absence of radiotracer uptake at the corresponding level (*arrow*). These lesions remained stable during follow-up

On US, the segmental form appears as non-communicating multiple cysts and dysplastic stroma confined to a single renal pole. Due to its imaging resemblance to multilocular cystic nephroma and cystic partially differentiated nephroblastoma, further evaluation may be needed. MRI and CT reveal a multiseptated cystic mass (Fig. 4). Radiologic evaluation is not always sufficient for definitive diagnosis. In such cases, renal scintigraphy with dimercaptosuccinic acid (DMSA) is valuable in assessing function of segmental multicystic dysplastic areas [16, 18]. If the imaging remains inconclusive, biopsy or surgical excision may be required to exclude malignancy.

### Infectious and granulomatous renal pseudotumors

#### Focal bacterial nephritis and renal abscess

**Fig. 5:**
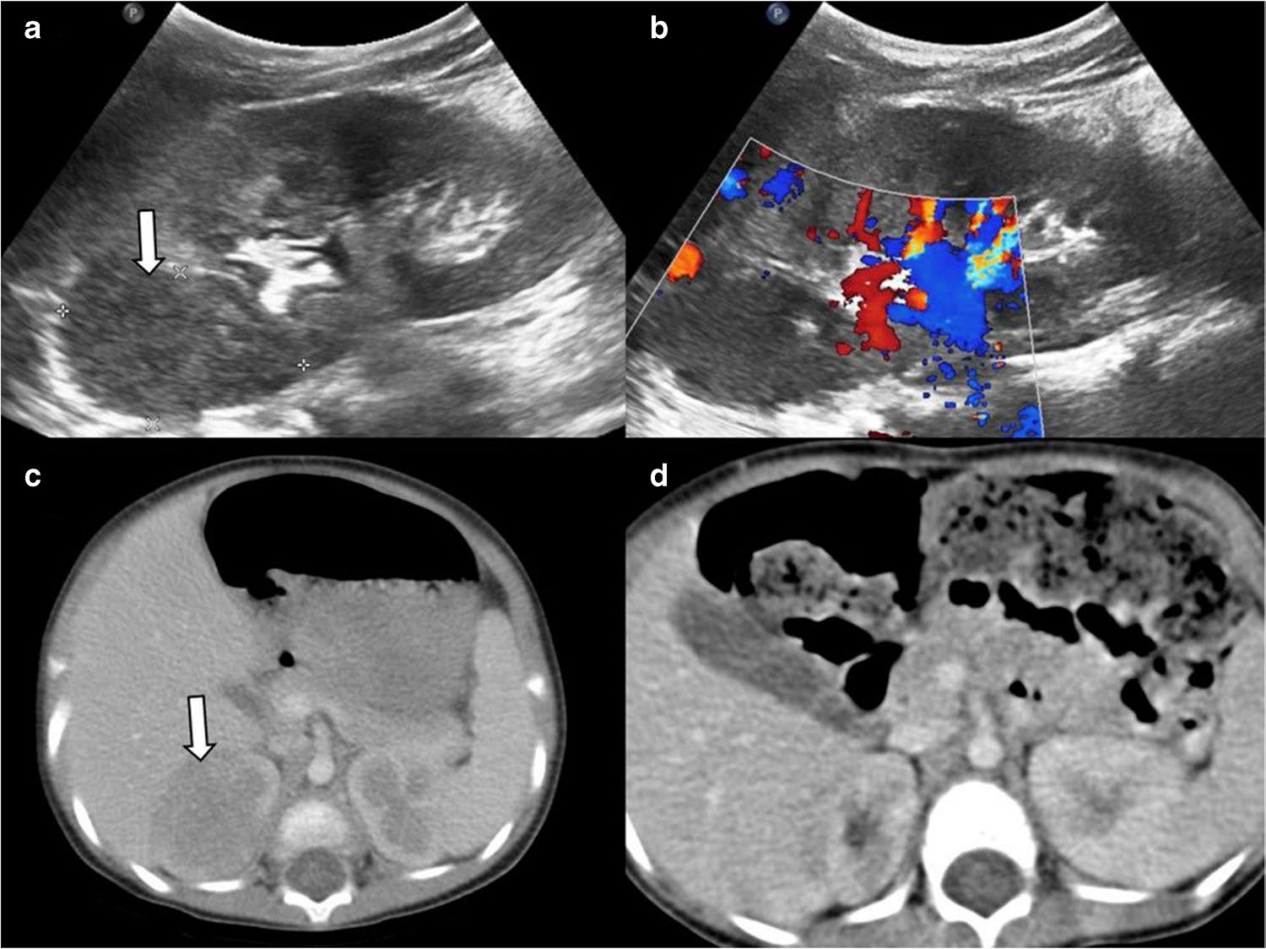
Acute focal bacterial nephritis. Two girls aged 6 years (**a**,** b**) and 7 years (**c**,** d**) were admitted to hospital with fever and abdominal pain and they had also leukocytosis. **a**,** b** Longitudinal grayscale ultrasound (US) image (**a**) demonstrates a hypoechoic lesion in the upper pole of the left kidney (*arrow*), with no vascularization on color Doppler US (**b**). The lesion showed resolution with antibiotic therapy (not shown). **c** Axial contrast-enhanced computed tomography (CT) image shows an ill-defined hypodense lesion (*arrow*) in the upper pole of the right kidney, mimicking a mass. **d** Three months later, axial contrast-enhanced CT image performed due to suspicion of ileus shows regression of the previously detected hypodense lesion

Acute focal bacterial nephritis is a localized, non-liquefactive bacterial infection of the kidney affecting one or more lobes [19]. Solitary lesions can resemble Wilms’ tumor or renal cell carcinoma, while multifocal lesions may mimic lymphoma or metastases. Characteristic US findings include hypo- or hyperechoic focal lesions with poorly defined margins, focal loss of corticomedullary differentiation, and nephromegaly [19, 20]. The sensitivity of US in detecting focal bacterial nephritis is limited. Focal bacterial nephritis typically appears as an ill-defined hypodense lesion on CT [19] (Fig. 5). CEUS or MRI may serve as alternative imaging modalities in pediatric patients [20, 21]. Diffusion-weighted imaging (DWI) is particularly useful, as lesions demonstrate restricted diffusion and lower apparent diffusion coefficient (ADC) values compared to normal renal parenchyma. It has been noted that the sensitivity of DWI in diagnosis is higher compared to CT [22]. Clinical and laboratory findings, including fever, pyuria, flank pain, positive urine culture, and leukocytosis, are important for distinguishing focal bacterial nephritis from neoplasm, and regression of the lesion is expected with appropriate treatment. Imaging features such as perirenal fat stranding, Gerota’s fascia thickening and diffuse nephromegaly support the diagnosis of infection.

**Fig. 6:**
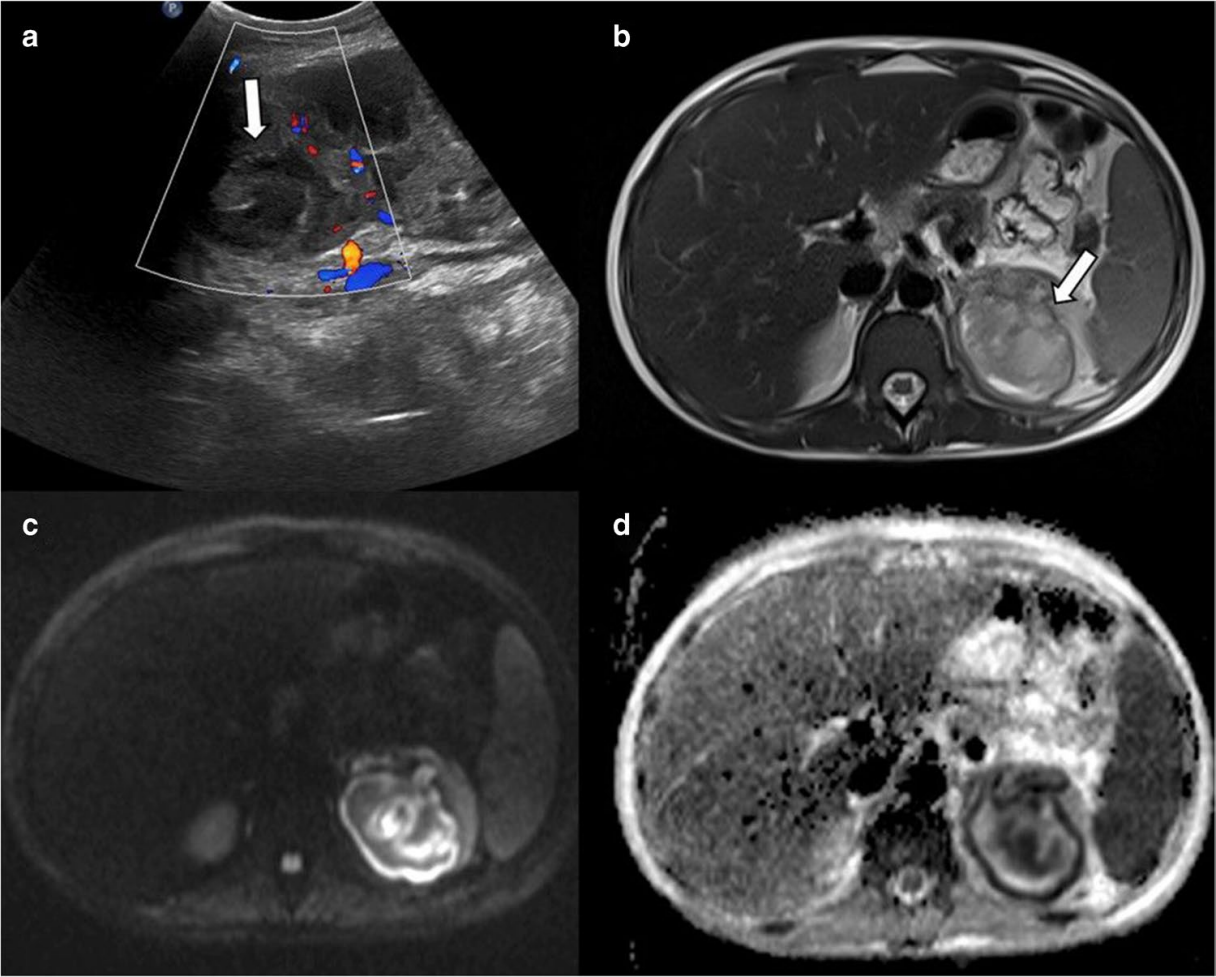
Renal abscess. A 7-year-old girl had fever and left upper quadrant pain. **a** Longitudinal ultrasound (US) image demonstrates a heterogeneous hypoechoic lesion in the upper pole of the left kidney, with no internal vascularity on color Doppler US (*arrow*). **b** Axial T2-weighted image reveals a mildly hyperintense lesion with a hypointense rim in the same region (*arrow*). **c**,** d** Diffusion-weighted imaging (*b* = 800 s/mm^2^) (**c**) and the apparent diffusion coefficient map (**d**) show restricted diffusion in the center and wall of the lesion, consistent with an abscess. The lesion was aspirated and *Stenotrophomonas maltophilia* was identified on the culture

Renal abscess is a complication of focal pyelonephritis and may be parenchymal or perinephric. In the early stages, abscesses appear as ill-defined hypovascular areas [23]. A mature abscess presents as a well-defined complex cystic lesion with a thick, irregular wall. On US, a typical abscess appears as a heterogeneous mass with posterior acoustic enhancement and absent internal flow on color Doppler. These features can also be seen in necrotic malignant tumors. The patient’s symptoms and laboratory findings are crucial in differential diagnosis. If clinical findings are inconsistent with an abscess, MRI may be useful for confirming the diagnosis. Abscesses appear hypointense on T1- and hyperintense on T2-weighted MRI. They may exhibit perilesional edema and fluid–fluid levels. Pseudocapsules may enhance in the venous phase. Restricted diffusion is typically observed at the center of the abscess, with ADC values lower than focal nephritis or sterile fluid collections. On CT, they appear as round or geographic hypodense lesions with enhancing thick wall (Fig. 6) [22, 24, 25]. The wall of Wilms’ tumor is typically more irregular and the presence of fat stranding may be particularly helpful in distinguishing between an abscess and a Wilms’ tumor.

#### Renal tuberculosis

Tuberculosis is caused by the *Mycobacterium tuberculosis* bacterium. Human immunodeficiency virus infection and immunodeficiency disorders are well-recognized risk factors for tuberculosis [26]. Urogenital tuberculosis is the most common form of extrapulmonary tuberculosis [27]. Renal tuberculosis can affect both the parenchyma and the collecting system. Early findings include papillary necrosis and small (< 3 mm) granulomas with minimal or no enhancement. Renal tuberculosis can present as one or more solid parenchymal masses without the involvement of the urinary tract, referred to as the pseudotumoral type. These lesions often show peripheral enhancement and may mimic metastases, lymphoma, Wilms’ tumor, and renal cell carcinoma. Tuberculous abscesses may appear as multilocular cystic lesions, mimicking Bosniak IIF/III cysts. In cases of pseudotumoral tuberculosis, imaging is usually insufficient for differential diagnosis, necessitating biopsy [28, 29].

#### Xanthogranulomatous pyelonephritis

Xanthogranulomatous pyelonephritis is an aggressive variant of chronic pyelonephritis characterized by renal parenchymal destruction and replacement with granulomatous tissue containing lipid-laden macrophages. It may sporadically occur in children. It is believed that chronic obstruction and infection are the primary initiating factors [30]. Obstruction is often caused by stones, which can serve as a nidus for infection. Staghorn stones are observed in approximately 80% of cases [31]. Clinical findings include flank pain, abdominal mass, and weight loss. Urine culture is positive in 70% of patients [32].

Focal xanthogranulomatous pyelonephritis affects one segment of the kidney or one pole if there is a duplex collecting system. It appears as a localized hypoechoic mass on US, often without an obstructing stone. The intensity of the solid component of the lesion on T1-weighted MRI varies according to the amount of xanthoma cells and is typically iso- to hyperintense relative to renal parenchyma. On T2-weighted MRI, the lesion often appears hypointense relative to fluids due to high proteinaceous content. Although CT is less commonly preferred for diagnosis in children, focal xanthogranulomatous pyelonephritis appears as a well-defined hypodense intrarenal mass [32].

**Fig. 7:**
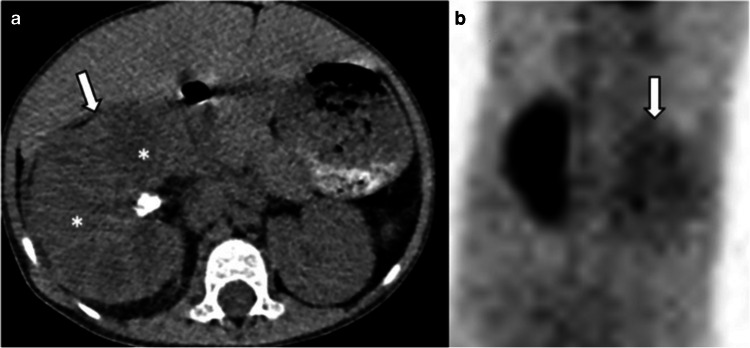
Xanthogranulomatous pyelonephritis. A 7-year-old girl was admitted to the hospital with right flank pain and vomiting. **a** Axial non-contrast computed tomography image shows a right pelvic stone, enlarged right kidney (*arrow*), and bear’s paw sign (*asterisk*). **b** Scintigraphy with 99mTc-diethylene-triamine-pentaacetate demonstrates severely decreased function of the right kidney, with a relative contribution of only 10% (*arrow*)

In diffuse xanthogranulomatous pyelonephritis, the entire kidney enlarges but maintains its reniform shape. Ultrasound shows nephrolithiasis and multiple hypoechoic areas. CT reveals global kidney enlargement, staghorn stone, hypodense rounded areas replacing the renal parenchyma, and perirenal inflammation. Hypodense areas represent dilated calyces and focal destruction areas filled with pus or debris. The combination of multiple hypodense areas and accompanying rim enhancement creates a characteristic “bear’s paw sign” (Fig. 7). The absence of staghorn stones does not rule out xanthogranulomatous pyelonephritis. On MRI, the solid portions of the lesion appear iso- to hyperintense on T1-weighted images and iso- to hypointense on T2-weighted images relative to the renal parenchyma due to the accumulation of xanthoma cells. The cavities and abscesses appear hypointense on T1-weighted images and hyperintense on T2-weighted images. Contrast-enhanced T1-weighted MRI shows rim enhancement of collections with non-enhancing internal contents. Scintigraphy with 99mTc-diethylene-triamine-pentaacetate or mercaptoacetyltriglycine reveals non-functioning kidneys in approximately 80% of diffuse cases [30, 32].

Focal xanthogranulomatous pyelonephritis can mimic Wilms’ tumor, renal cell carcinoma, and lymphoma. Due to nonspecific imaging findings, differential diagnosis often requires biopsy. Although diffuse xanthogranulomatous pyelonephritis can be diagnosed preoperatively based on typical imaging findings, pathological confirmation is needed in atypical infiltrative cases [32].

#### Renal sarcoidosis

Sarcoidosis is a chronic, idiopathic systemic inflammatory disease characterized by non-caseating granulomas. Although it is more commonly seen in adults aged 25–45, pediatric cases are rare, with an estimated incidence of 0.6–1.02 per 100,000 [33, 34]. Pulmonary involvement is typical, whereas abdominal findings are less frequent [35].

It can present as nephrotic syndrome, glomerulonephritis, tubulointerstitial nephritis, and rarely renal failure. Due to expression of 1-alpha hydroxylase by activated macrophages in sarcoidosis granulomas, vitamin D and calcium metabolism are abnormal in these patients. This can lead to hypercalcemia and hypercalciuria resulting in nephrocalcinosis, nephrolithiasis, and interstitial calcium deposition [36].

Renal sarcoidosis may rarely present as solitary or multiple pseudotumoral lesions that can mimic Wilms’ tumor, renal cell carcinoma, lymphoma, metastases, or oncocytoma. Pseudotumoral renal sarcoidosis may appear as hypo- or hyperechoic lesions on US. Lesions can be homogeneous or mildly heterogeneous on pre-contrast T1- and T2-weighted MRI. On contrast-enhanced MRI, they demonstrate less enhancement compared to the normal renal cortex. These lesions may appear hypo-, iso-, or hyperdense compared to the normal renal parenchyma on non-contrast CT but show hypoenhancement on contrast-enhanced CT [37]. Given the nonspecific nature of imaging findings, clinical presentation and laboratory findings are essential for diagnosis, and biopsy is usually required to establish a definitive diagnosis [35].

#### Renal hydatid cyst

Hydatid disease is a parasitic infection caused by the larva of *Echinococcus* tapeworm. This disease is most commonly caused by *Echinococcus granulosus*, whereas *Echinococcus multilocularis*, though less common, is associated with a more aggressive clinical course. The most commonly involved organ is the liver, although other organs are sometimes infected through hematogenous dissemination. Renal involvement occurs in approximately 3% of cases. Although usually asymptomatic, the most common clinical signs are pain, dysuria, and flank mass [38]. Hydatiduria is a pathognomonic sign and indicates the rupture of the cyst. X-ray may demonstrate thin arc-shaped calcifications which are characteristic of hydatid cysts. The serological testing may assist in establishing the diagnosis [39].

Hydatid cysts are classified into four types based on imaging morphology. All types of hydatid cysts usually demonstrate a low signal intensity rim on T2-weighted MRI, likely corresponding to dense, fibrous pericyst. Type 1 is a unilocular cystic lesion without any internal architecture. On US, type 1 hydatid cysts look like Bosniak type 1 cysts. Hydatid cysts have a bilayered wall composed of a pericyst and a laminated membrane. With patient movement, hydatid sand may shift, producing the so-called *snowstorm appearance*. A combination of US findings and serological testing is usually sufficient for diagnosis. Type 1 cysts demonstrate the signal intensity of fluid on T1- and T2-weighted MR images [40, 41].

**Fig. 8:**
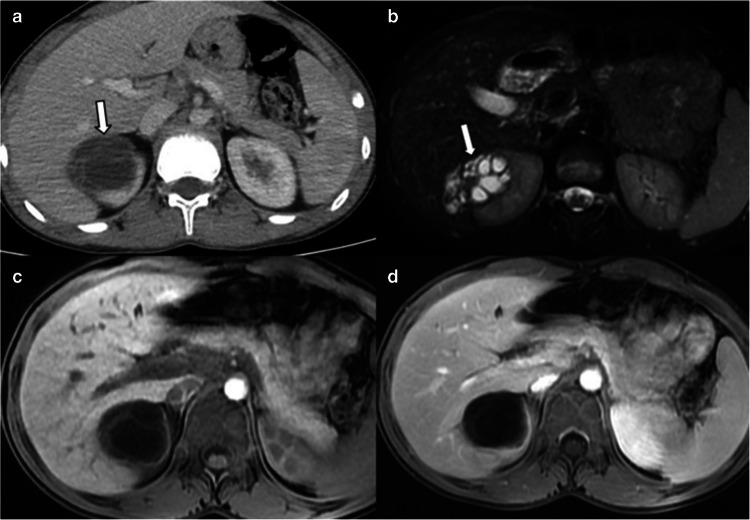
Type 2 renal hydatid cyst. A 15-year-old boy presented with right flank pain. **a** Axial contrast-enhanced computed tomography image reveals a multiseptated cystic lesion (*arrow*) in the upper pole of the right kidney, mimicking a neoplasm. **b** Axial fat-saturated T2-weighted image shows a multiloculated cystic lesion containing cysts of various sizes, compatible with daughter cysts (*arrow*). **c**,** d** Axial pre-contrast (**c**) and post-contrast (**d**) T1-weighted images demonstrate no internal vascularity. Serological tests were positive, and the lesion was treated by interventional radiology

Type 2 is a multiseptated cystic lesion with daughter cysts (Fig. 8). These lesions may mimic cystic partially differentiated nephroblastoma, multilocular cystic nephroma, and renal cell carcinoma. Detachment of endocyst from pericyst results in a floating membrane appearance on US. Multiple daughter cysts are separated by a maternal matrix containing membranes of broken cysts, scolices, and hydatid sand. This appearance is similar to a spoke-wheel pattern. Detached membranes are linear low signal-intensity structures on MRI regardless of sequence type. Daughter cysts have similar signal intensity to fluid on MRI and are hypodense compared to maternal matrix on CT. The maternal matrix appears hypointense on T1-weighted images and hyperintense on T2-weighted images. The wall and internal septae may show enhancement on CT and MRI [40, 41]. Key imaging findings for differential diagnosis include a T2 hypointense rim, the presence of detached membranes, and daughter cysts.

Type 3 refers to a completely calcified cyst, indicative of parasite death. These lesions appear as bright echogenic foci with posterior acoustic shadowing on US. Type 4 refers to a complicated cyst. Complications are rupture and superinfection, which may be seen in both type 1 and type 2 [40, 41].

### Vascular renal pseudotumors

#### Renal extramedullary hematopoiesis

Extramedullary hematopoiesis is the development of hematopoietic tissue outside the bone marrow. It generally occurs in the reticuloendothelial system, such as the liver, spleen, and lymph nodes. Extramedullary hematopoiesis is observed in patients who have hematologic disorders affecting the bone marrow. Renal involvement is very rare and may be parenchymal, parapelvic, or perirenal [42]. Parenchymal involvement can mimic Wilms’ tumor, renal cell carcinoma, and lymphoma. Parapelvic and perirenal lesions are often bilateral, and these lesions are similar to lymphoma.

Extramedullary hematopoiesis typically appears as homogeneous, hypovascular, enhancing soft tissue masses on imaging [43–45]. Chemical shift MRI can demonstrate microscopic fat within the lesion. Extramedullary hematopoietic foci containing reticuloendothelial cells can be visualized using Tc-99m sulfur colloid due to their tracer uptake properties.

Although the clinical history of the patient may suggest extramedullary hematopoiesis in the differential diagnosis, biopsy is often required due to the nonspecific imaging findings [43, 44]. In patients with known myeloproliferative disorders, extramedullary hematopoiesis should be considered prior to biopsy, as such lesions may carry an increased risk of bleeding due to their high vascularity.

#### Renal arteriovenous malformation

Renal arteriovenous malformations are abnormal connections between the arterial and venous systems of the kidneys via a nidus composed of multiple dilated and tortuous vessels [46]. They are uncommon, with a prevalence of approximately 0.04% in the general population [47].

Arteriovenous malformations appear as lobulated hypoechoic or anechoic structures on US, potentially mimicking cystic tumors. If not evaluated with color Doppler US, patients may be referred for MRI or CT due to suspicion of a mass. In the venous phase of contrast-enhanced MRI or CT, they may resemble renal cell carcinoma. Therefore, in the presence of a unilocular or multilocular cystic renal lesion on US, evaluation with color Doppler US is crucial. A mosaic flow pattern within the cystic lesion and speckling in the surrounding soft tissue due to high-velocity flow can be observed on color Doppler US. Spectral Doppler analysis may demonstrate increased flow velocity, decreased arterial resistance, and arterial waveforms in the renal vein.

**Fig. 9:**
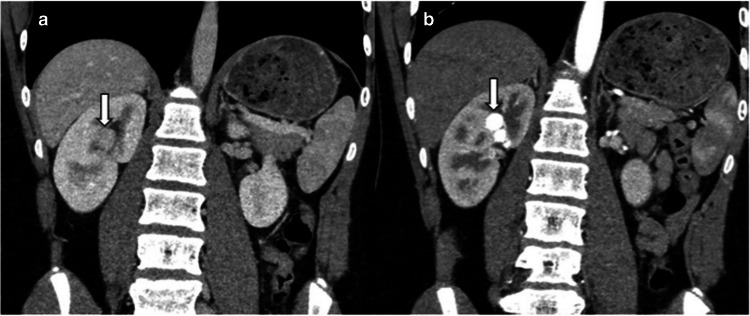
Renal arteriovenous malformation. A 16-year-old boy was admitted to the hospital with hematuria. **a** Coronal venous phase contrast-enhanced computed tomography (CT) image shows a nodular isodense lesion (*arrow*) in the right renal sinus, mimicking a mass. **b** Coronal arterial phase contrast-enhanced CT image demonstrates tortuous vascular structures in the right renal sinus with attenuation similar to the aorta, consistent with an arteriovenous malformation (*arrow*). The lesion was treated via embolization in the Department of Interventional Radiology

MRI shows flow voids in the lesion due to the high flow nature of the lesion. Three-dimensional contrast-enhanced MR angiography can be useful in demonstrating the feeding artery, similar enhancement with the aorta in the abnormal tortuous vascular structures of the nidus, and early opacification of the renal vein. CT angiography is commonly used to confirm the diagnosis, detect the feeding arteries, and assess the extent of arteriovenous malformation (Fig. 9) [46, 48].

#### Spontaneous subcapsular hematoma

**Fig. 10:**
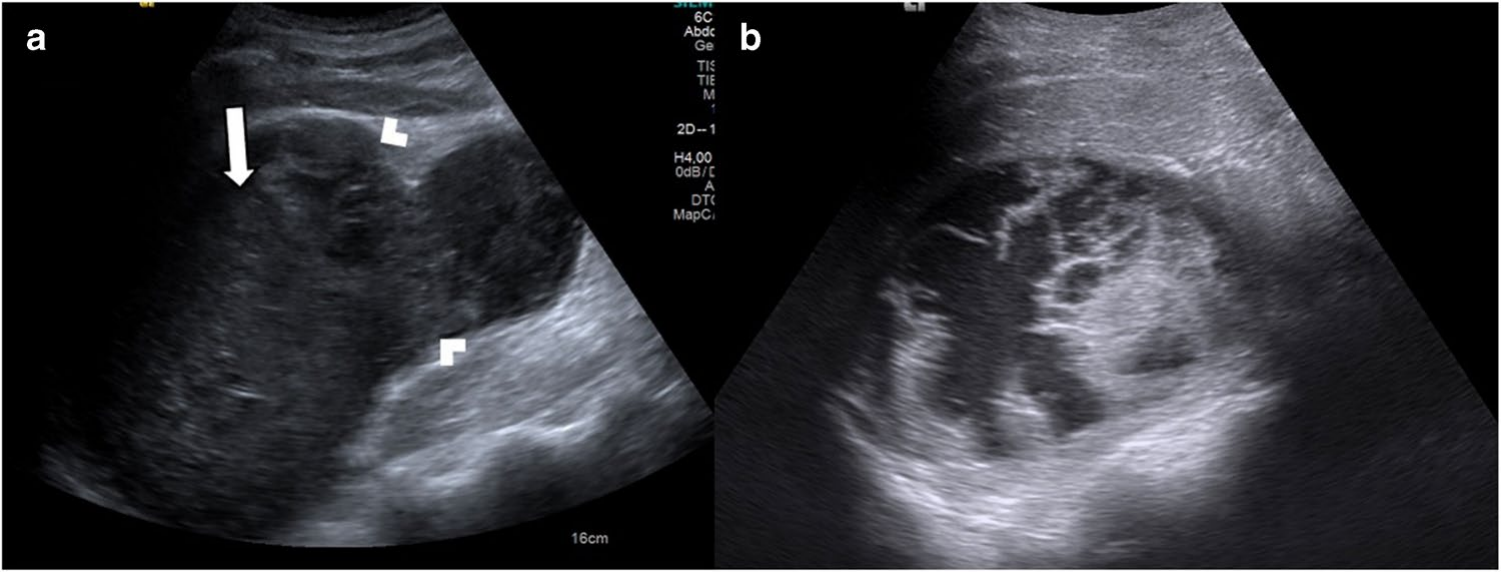
Spontaneous subcapsular hematoma. A 15-year-old boy presented to the Emergency Department with acute left flank pain. **a** Axial grayscale ultrasound (US) image reveals a mildly heterogeneous solid mass (*arrow*) compressing the left renal parenchyma (*arrowheads*). Color Doppler US revealed no vascularity within the lesion (not shown). **b** Follow-up axial grayscale US image performed 2 months later demonstrates a decrease in the lesion size with partial liquefaction. The underlying etiology remained unclear

Spontaneous subcapsular renal hematoma is a rare entity [49]. Patients may present with back or flank pain, and tenderness. Possible etiologies in children include tumors, vascular diseases, infections, and the use of anticoagulant/antiplatelet drugs, with a small proportion of cases being idiopathic [50, 51]. It has been shown that hypertension is a risk factor in some cases where the etiology cannot be determined [52]. The diagnosis of liquefied or layered hematomas is straightforward with US. However, these lesions may present as heterogeneous subcapsular solid masses, potentially mimicking neoplasms (Fig. 10). The absence of internal vascularity on color Doppler US is an important diagnostic feature for hematoma. In cases of large lesions or diagnostic uncertainty, further imaging is often required to confirm the diagnosis and determine the underlying etiology [53]. In emergency settings, CT is preferred due to its rapid acquisition time, offering high sensitivity—approaching 100%—for hematoma detection. MRI is appropriate for both diagnostic and etiological evaluation in pediatric patients [52, 54, 55].

### Miscellaneous renal pseudotumors

#### Scarred kidney and regeneration nodule

Normal renal tissue can undergo hypertrophy and hyperplasia. In scarred kidneys, focal nodular compensatory hypertrophy can develop in relatively preserved renal parenchyma, which is referred to as regeneration nodule [56–58]. Regeneration nodules can cause outer renal contour lobulation or compress the pelvicalyceal system, mimicking multifocal Wilms’ tumor, metastases, or lymphoma. Sometimes, spared parenchyma between scarred areas may appear like a mass. In these cases, it may be possible to rule out a mass with the help of corticomedullary differentiation on contrast-enhanced CT or MRI.

**Fig. 11:**
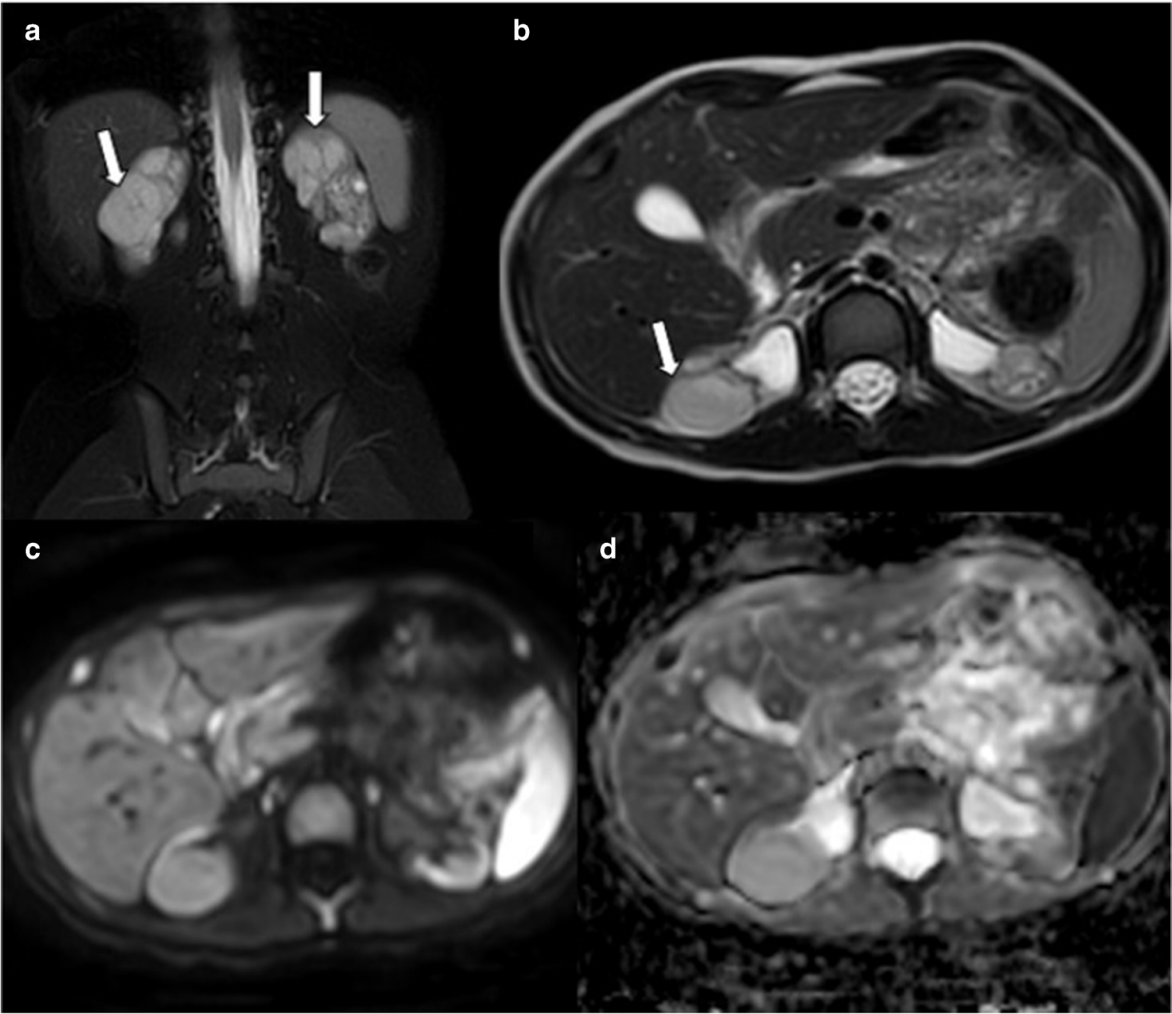
Scarred kidney and regeneration nodules. A 6-year-old boy undergoing ultrasound follow-up for chronic kidney disease was found to have multiple lesions in both kidneys.** a**,** b** Coronal fat-saturated (**a**) and axial non-fat-saturated (**b**) T2-weighted images show multiple bilateral hyperintense nodular lesions (*arrows*) located between scarred atrophic renal tissues. Note that these lesions have homogeneous signal intensity. **c**,** d** Diffusion-weighted imaging (*b* = 600 s/mm^2^) (**c**) and apparent diffusion coefficient map (**d**) demonstrate no restricted diffusion in the lesions, consistent with regeneration nodules

In children with chronic kidney disease, the presence of multiple nodular lesions on US necessitates consideration of regeneration nodules in the differential diagnosis. Due to impaired renal function, contrast agents are generally avoided, making differential diagnosis challenging when relying on non-contrast CT and MRI. In such cases, biopsy may be required. To avoid the biopsy, DMSA SPECT or DWI can be utilized. DMSA SPECT is a non-toxic, non-invasive modality for renal cortical imaging. DMSA is taken up by functional nephrons. Regeneration nodules typically demonstrate normal or increased uptake, whereas DMSA uptake is reduced or absent in neoplasms [57]. DWI reveals heterogeneous restricted diffusion in neoplasms, whereas regeneration nodules do not show restricted diffusion and have higher ADC values compared to the surrounding scarred tissue (Fig. 11) [56].

#### Hemorrhagic renal cyst

Renal cysts are rare in children with an incidence of less than 0.5% [59]. It can be a simple or complicated renal cyst. In case of a simple renal cyst, differentiation from renal neoplasms is usually straightforward. Renal cysts do not show any enhancement on dynamic CT or MRI. Complicated cysts can be due to infection or hemorrhage. Hemorrhagic cysts may exhibit features such as wall thickening, septations, solid components, and mural nodularity, which can mimic neoplastic lesions [60].

**Fig. 12:**
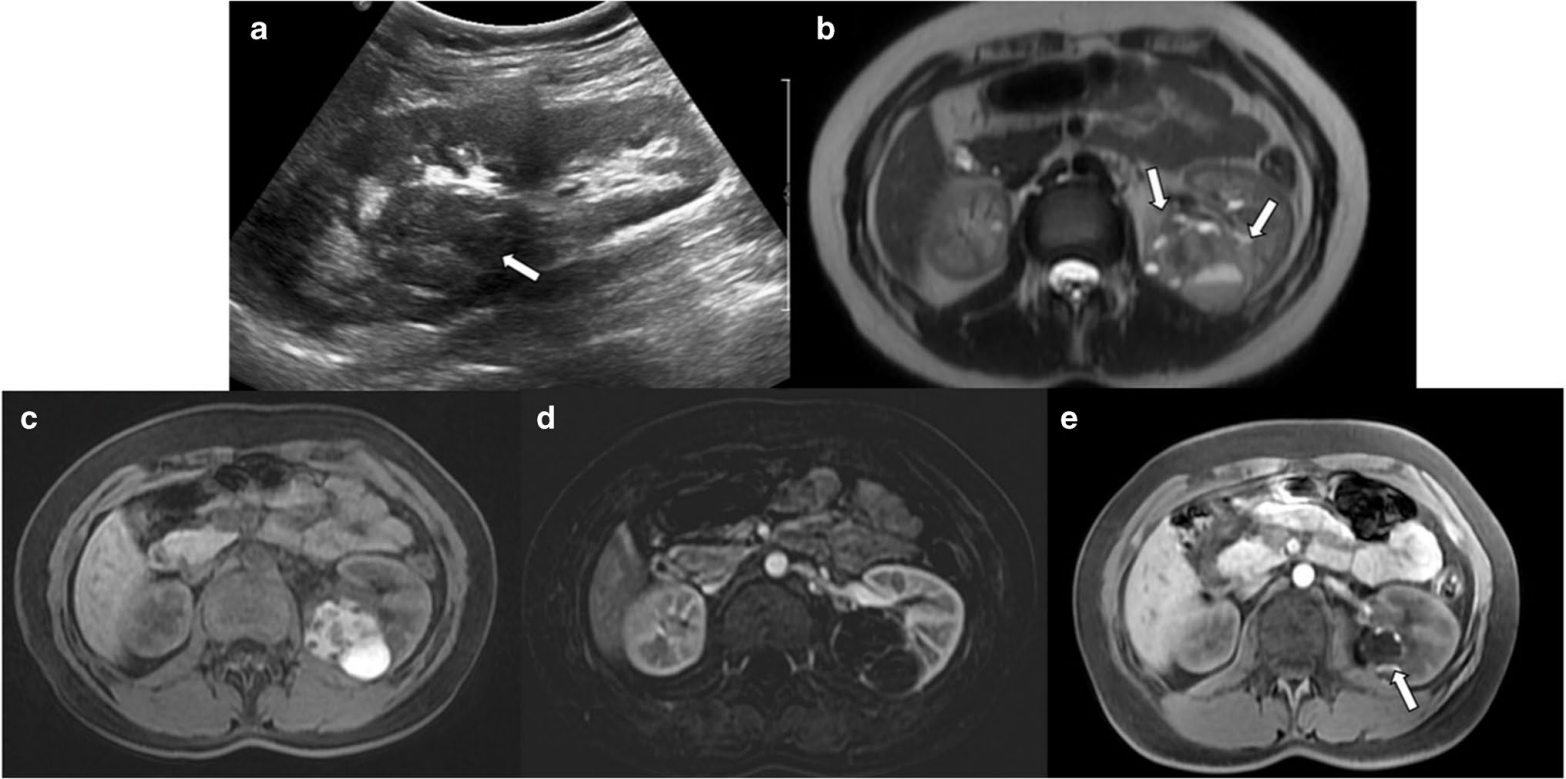
Hemorrhagic cyst. A 17-year-old boy was admitted to the Emergency Department with sudden onset left flank pain. **a** Longitudinal grayscale ultrasound image of the left kidney demonstrates a heterogeneous lesion (*arrow*) extending from the renal sinus to the capsule. **b** Axial T2-weighted image shows a lesion containing some cystic areas and fluid–fluid level (*arrows*) in the left kidney. **c** Axial fat-suppressed pre-contrast T1-weighted image reveals that the lesion is predominantly hyperintense, consistent with hemorrhagic content. **d** Axial post-contrast substraction image demonstrates only septal enhancement without any solid component, consistent with hemorrhagic cyst. **e** Follow-up axial fat-suppressed pre-contrast T1-weighted image obtained 3 months later shows a decrease in the lesion size and regression of most of the hemorrhagic content (*arrow*)

On US, they may appear as completely solid or mixed solid-cystic masses. The absence of internal vascularity on color Doppler US is a crucial diagnostic feature. However, if the lesion appears entirely solid or demonstrates suspicious vascularity, it may mimic malignant tumors. In such cases, MRI is valuable for diagnostic confirmation. T1 hyperintensity on MRI is a typical feature for hemorrhagic cysts, but it is also seen in hemorrhagic renal tumors. Hemorrhagic cysts can show thin peripheral wall enhancement. Enhancement of the irregular wall or solid component is suggestive of malignancy. Therefore, subtraction images from dynamic contrast-enhanced MRI are necessary to detect if there is any enhancing solid component (Fig. 12).

When contrast-enhanced images are not available, it has been observed that the T1-weighted lesion-to-muscle signal intensity ratio can be used to differentiate between hemorrhagic cysts and neoplasms (optimal cut-off value 1.39). Hemorrhagic cysts have been shown to exhibit higher signal intensity ratio compared to malignant tumors. Homogeneous renal lesions with an attenuation of ≥ 70 Hounsfield units on non-contrast CT are highly suggestive of high-attenuation hemorrhagic cysts, with a diagnostic accuracy exceeding 99.9%, rather than malignancy [59–62].

## Conclusion

Renal pseudotumors in children represent a diverse group of non-neoplastic lesions that can mimic malignant tumors on imaging. In pediatric patients, imaging modalities that do not involve ionizing radiation should be prioritized. Ultrasound is usually the initial imaging modality due to its accessibility, safety, and effectiveness. MRI can be used as the second-line imaging modality when a definitive diagnosis cannot be made with US. Familiarity with the imaging findings of pseudotumors and their evaluation in conjunction with clinical and laboratory findings is crucial for differential diagnosis and prevention of unnecessary invasive procedures.

## Data Availability

No datasets were generated or analysed during the current study.
